# Transcriptomic analysis of human endometrial stromal cells during early embryo invasion

**DOI:** 10.1080/07853890.2021.1988139

**Published:** 2021-10-13

**Authors:** Shuo Han, Minghui Liu, Shan Liu, Yuan Li

**Affiliations:** Medical Center for Human Reproduction, Beijing Chao-Yang Hospital, Capital Medical University, Beijing, China

**Keywords:** Endometrial stromal cells, embryo invasion, transcriptome

## Abstract

**Purpose:**

During early embryo invasion (48 h after embryo attachment), what functional changes accompany dynamic gene expression alterations in human endometrial stromal cells?

**Method:**

In the present study, primary human endometrial stromal cells (phESCs) were cultured. After *in vitro* decidualization, primary human endometrial stromal cells (phESCs) were cultured with blastocysts for 48 h. During this process, blastocysts attached and invaded the phESCs (embryo-invaded primary human endometrial stromal cells, ehESCs). We performed comprehensive transcriptomic profiling of phESCs (two replicates) and ehESCs (five replicates) and analyzed the differentially expressed gene (DEGs) sets for gene ontology (GO) terms and Kyoto encyclopaedia of genes and genomes (KEGG) pathway enrichment. To analyse potential connectivity patterns between the transcripts in these DEG sets, a protein-protein interaction (PPI) network was constructed using the STRING database.

**Results:**

A total of 592 DEGs were identified between phESCs and ehESCs after embryo invasion. Primary human endometrial stromal cells underwent significant transcriptomic changes that occur in a stepwise fashion. Oxidative phosphorylation, mitochondrial organization, and P53 signalling pathways were significantly altered in phESCs after embryo invasion. EP300 may play a key role in regulating transcription *via* chromatin remodelling to facilitate the adaptive gene expression changes that occur during embryo invasion.

**Conclusions:**

Our data identify dynamic transcriptome changes that occur in endometrial stromal cells within 48 h after embryo invasion. The pathways that we found to be enriched in phESCs after embryo invasion (oxidative phosphorylation, mitochondrial organization, and P53 signalling) may represent novel mechanisms underlying embryo implantation, and may illuminate the reasons that some women experience reproductive failure.

**Key messages**
- Human endometrial stromal cells have undergone changes in gene expression regulation and signalling pathways during the embryo invasion.
- Mitochondrial-oxidative phosphorylation changes in human stromal cells manifested as down-regulation of gene expression in the electron transport chain.
- TP53 signalling pathway and transcriptional regulator EP300 assist stromal cells to get adaptive changes during embryo invasion phase.

## Introduction

Successful embryo implantation is required for a successful pregnancy. Embryo implantation depends on a highly coordinated process occurring between a viable embryo and a receptive endometrium [1,2]. Identifying the regulatory mechanisms that occur within endometrial stromal cells during the embryo invasion stage would help uncover ways to improve impaired endometrial receptivity and to decrease unfavourable pregnancy outcomes among women experiencing reproductive failure. However, the genetic and transcriptomic changes that occur within human endometrial stromal cells during embryo invasion are poorly understood, in no small part due to the difficulty and ethical factors involved with evaluating gene regulation in endometrial stromal cells during embryo invasion in human subjects.

The endometrium begins to decidualize before embryo attachment [3]. In the clinic, endometrial receptivity assays (ERA) are performed to improve the success of embryo implantation [4]. However, ERA does not perform well in identifying patients that may experience recurrent implantation failures [5,6]. During IVF, after embryo attachment and invasion into the endometrium, endometrial stromal cells undergo dynamic changes in gene expression to help facilitate the survival of the embryo [7–9]. Therefore, there are likely important pathways that are regulated in endometrial stromal cells during the embryo invasion stage. These important genes and pathways could be potential biomarkers that may be used to predict implantation outcomes and to better help facilitate our understanding of the key mechanism of successful embryo implantation, ultimately leading to improved implantation rates. Transcription factors, including C/EBP, FOXO1, CREB, and STAT5 are involved in decidualization [10–14]. The mTORC1/P21/COX2 pathway is involved in endometrial decidualization and is related to senescence [15–17]. Nevertheless, it is unknown whether these endometrial receptivity/decidualization-related genes function to facilitate embryo invasion.

Two main methods have been employed to investigate the embryo invasion period (48 h): one method is an animal model used to explore molecular mechanisms *in vivo* [18–20]; the other is an *in vitro* human embryo invasion model that uses a conditioned medium or villous trophoblasts in co-culture with endometrial stromal cells [21–23]. There are flaws in these two methods. First, there is great variability between species, especially regarding the time of embryo implantation and decidualization, and the rate of embryonic development. Animal models do not accurately recapitulate the process of embryo implantation in humans. Second, using villous trophoblasts does not truly reflect the signal feedback of human endometrial stromal cells, because of the great differences between villous trophoblasts and embryos.

To explore the complex interactions between the embryo and the endometrium, the most suitable endometrial stromal sample is from the human body at the time that the embryo has just invaded. Presently, the earliest time to accurately detect pregnancy is 5 days after embryo transfer, which can be detected by serum hCG levels after 4–5 days have passed since embryo implantation. At this time, embryo and endometrial stromal cells have finished the primary signal crosstalk, and the stromal cells have entered into the stages of angiogenesis and cell proliferation [8,24–26]. Therefore, an *in vitro* model including embryos and primary stromal cells may be a better way to study the embryo invasion stage. In the past, there were many *in vitro* models of embryo implantation, including different cell sources and different culture systems, according to different purposes. Cell sources include stromal cells, epithelial cells, natural killer cells, villous trophoblast cells, and human/animal embryos. The culture systems include methods of 2-dimensional culture, trans-well culture, and 3-dimensional culture [22,27]. To more adequately explore changes of key genes and signalling pathways within endometrial stromal cells during embryo invasion (48 h after embryo attachment), we established an *in vitro* model using human embryos and primary stromal cells.

We aimed to identify the dynamic gene regulation that occurs within human endometrial stromal cells (hESCs) during the embryo invasion stage (48 h after embryo attachment). We used an *in vitro* model consisting of primary human endometrial stromal cells (phESCs) and blastocysts to analyze the endometrial transcriptome patterns in hESCs after embryos had invaded for 48 h. Additionally, we aimed to identify key pathways and genes that may regulate phESC-embryo interactions. Our findings may provide a foundation for targeted studies on embryo implantation and women experiencing reproductive failure, and may ultimately improve IVF outcomes.

## Material and methods

### Subject enrolment and endometrial biopsy

Human endometrial stromal cells were obtained from a single patient undergoing IVF after bilateral salpingectomy. The patient was 32 years old with regular menstrual cycles (28–32 days) and had no history of infectious diseases or genetic diseases. During the patient's natural cycles, we assigned the day of the urinary LH peak (hemtrue^®^, Shanghai, China) as LH + 0. Samples were collected in the late-proliferative phase (P group) and on day LH + 7 (N group). Biopsies were performed at the fundal part of the uterus under sterile conditions using a Pipelle catheter (Yajie^®^, Jiangxi, China). All biopsies were performed by the same surgeon. The endometrial receptivity array endometrial biopsy was performed in the luteal phase (LH + 5) of the menstrual cycle, before initiating the embryo transfer cycle. The blastocysts were obtained from a second patient, whose blastocysts were to be discarded because of poor quality. Both patients included in this study provided signed informed consent. This study was approved by the Ethics Review Board of Beijing Chao-Yang Hospital (2020-Science-279).

### Isolation and culture of endometrial stromal cells

To isolate endometrial stromal cells, a small amount of endometrial tissue was placed in a PBS solution containing 10% foetal bovine serum (FBS), washed twice to remove blood cells and mucus, and cut into 1–2 mm pieces using eye scissors. The samples were centrifuged, then digested with 0.25% trypsin plus 0.53 mM EDTA at 37 °C for 30 min; subsequently, the samples were passed through a 100 µm cell strainer (BD). The stromal cells passed through the cell strainer and were collected [28]. After centrifugation and a single wash, the stromal cells were resuspended in 10% FBS + DMEM/F12 medium and inoculated in a 35 mm dish. After 24 h, the dish was gently agitated to dislodge any non-adherent cells, the embryos did not float, and rather remained attached to the phESCs that were adhered to the surface of the dish.

### Embryo preparation

Discarded low-quality blastocysts were collected and frozen for this research study. In the experimental group, the grading of six poor-quality blastocysts (day 6) was 4CC, 4CC, 5CC, 5CC, 5CC, and 6CC, respectively, according to the score criteria described by Gardner. After thawing, the embryos were cultured in G2-plus (Vitrolife, Sweden) for 4 h, and then implanted in the cultured phESCs. The embryo freezing and thawing operations were performed as recommended in the KITAZATO reagent manual.

### Embryo invasion experiment

phESCs at *in vitro* passage 5 were used in all experiments in this study. To induce decidualization, phESCs were treated with 1 μM medroxyprogesterone acetate (MPA) and 0.5 mM 8-bromoadenosine 3′:5′-cyclic monophosphate (8-Br-cAMP) for 6 days. Then, A total of 5000 phESCs were seeded in 96-well plates. After 24 h culture, the stromal cell culture medium was replaced with human embryo culture medium G2-plus (Vitrolife, Sweden), and the thawed blastocysts were implanted on the phESCs layer, at one embryo per well [29]. After 48 h, embryonic cells were picked out, and the stromal cells were collected for RNA extraction and transcriptome sequencing.

### RNA extraction and library preparation

Total RNA was isolated using Trizol (Life Technologies) according to the manufacturer’s instructions. The NEBNext^®^ Ultra™ II RNA Library Prep Kit (New England Biolabs) for Illumina workflow was used to generate the sequencing libraries. The specific library construction process is described online at www.neb.com/products/e7770-nebnext-ultra-ii-rna-library-prep-kit-for-illumina.

### Quality assessment and quantification

We used the Qubit Fluorometer to determine the DNA concentration of the libraries and used a cut-off of >1.0 ng/μL to identify quality library preparation. A Qseq100 DNA analyzer was used to detect the DNA length distribution of the libraries. The criteria for qualification include the concentration of fragment length around 400 bp. We used the KAPA Library Quantification Kit to quantify the molar concentration of library DNA as a standard for library mixing. After the library was mixed and denatured, it was input into the Illumina HiSeq sequencing platform for high-throughput sequencing. Specific operations are described in the Illumina HiSeq instruction manual. During sequencing, both ends of the library were sequenced separately (i.e. paired-end, PE); therefore, each sample generated two data files: reads1 (R1) and reads2 (R2).

### Data processing and analysis

The raw data from the high-throughput sequencing was filtered to obtain high-quality data (CleanData). Subsequently, the clean data was compared with the designated reference genome (homo sapiens, grch37, annotation file obtained from Refgene) to calculate the efficiency of sequencing and the reference genome, and to evaluate the saturation of sequencing data and gene coverage. The quality assessment report before and after data processing was generated by FastQC. Sequencing data quality control and processing were as follows: (1) The BWA algorithm was used to remove low-quality regions at both ends of the sequence, using a mass threshold of 30; (2) removal of joint sequences; (3) removal of 5′ sequences containing fuzzy base N; (4) removal of sequences with length <60 bp (usually 10 bp).

### Construction of gene expression profiles

The quantification of RNA-seq data was performed by featureCounts (v2.0.0) using the default parameters (p -t exon -g gene_id). Gene expression profiles were constructed to quantify gene expression and to calculate gene expression in different samples. The distribution of gene expression values in each sample is described by box plot, density curve, and other illustrations. Genes were identified and classified, and included representation of RNA, rRNA, tRNA, Premier RNA, lncRNA, and novel genes.

### Identification of differentially expressed genes

The output from the featureCounts software was normalized by DESeq2 (v 1.30.1). DESeq2 normalization was performed among samples with different gene expression levels. Normalized counts were obtained from the raw counts, and used to compare gene expression between different groups. Specific methods were followed to perform Audic Claverie Test and Multiple Testing Correction: Benjamini Hochberg FDR; respective parameters: Maximum *p*-value cut-off: .05, fold-change cut-off: 2.0. Principle component analysis (PCA) was performed using the DESeq2-normalized data. Data visualization, including the generation of heatmaps and volcano plots, were performed using the R package “pheatmap” and “ggplot2”.

### Functional enrichment analysis

The differentially expressed genes were between the different sample groups were subjected to cluster analysis; volcano maps were generated for data visualization. GO/KEGG functional annotation and functional enrichment analysis of differential genes was performed to explore the functional and regulatory relationships of the differentially expressed genes. In all tests, completely known genes were set as the background, and *p*-values (i.e. EASE score) indicating significant overlap between various gene sets were calculated using Benjamini-corrected modified Fisher’s exact test. GO/KEGG-pathway terms associated with *p*-values <.05 were considered as significant, and only significant pathway terms are reported.

### Protein–protein interaction analysis

The protein–protein interaction (PPI) network for DEG-encoded proteins was performed using the STRING database. An interaction score >0.9 (highest confidence score) was considered significant, and PPI with a score >0.9 was visualized. The degree of a protein (node) equals the PPI interactions incident to the node in the whole network (https://string-db.org). To identify hub genes, the cytoHubba and Molecular Complex Deletion (MCODE) plug-ins for Cytoscape were employed, using the Maximal Clique Centrality (MCC) ranking method and significant network modules, with default parameters. Interaction networks of 10 hub genes were constructed using Cytoscape software.

### Quantitative real-time PCR

Quantitative real-time PCR (qRT-PCR) was employed to validate the results from high-throughput sequencing of the differentially expressed genes. PCR primer sequences used are listed in Table S1; primer specificity was confirmed on the NCBI Primer-BLAST website. RNA extraction, cDNA synthesis, and quantitative real-time PCR were performed according to previously reported methods [30].

## Results

### *In vitro* culture of phESCs and co-culture with embryos

**Figure 1.**
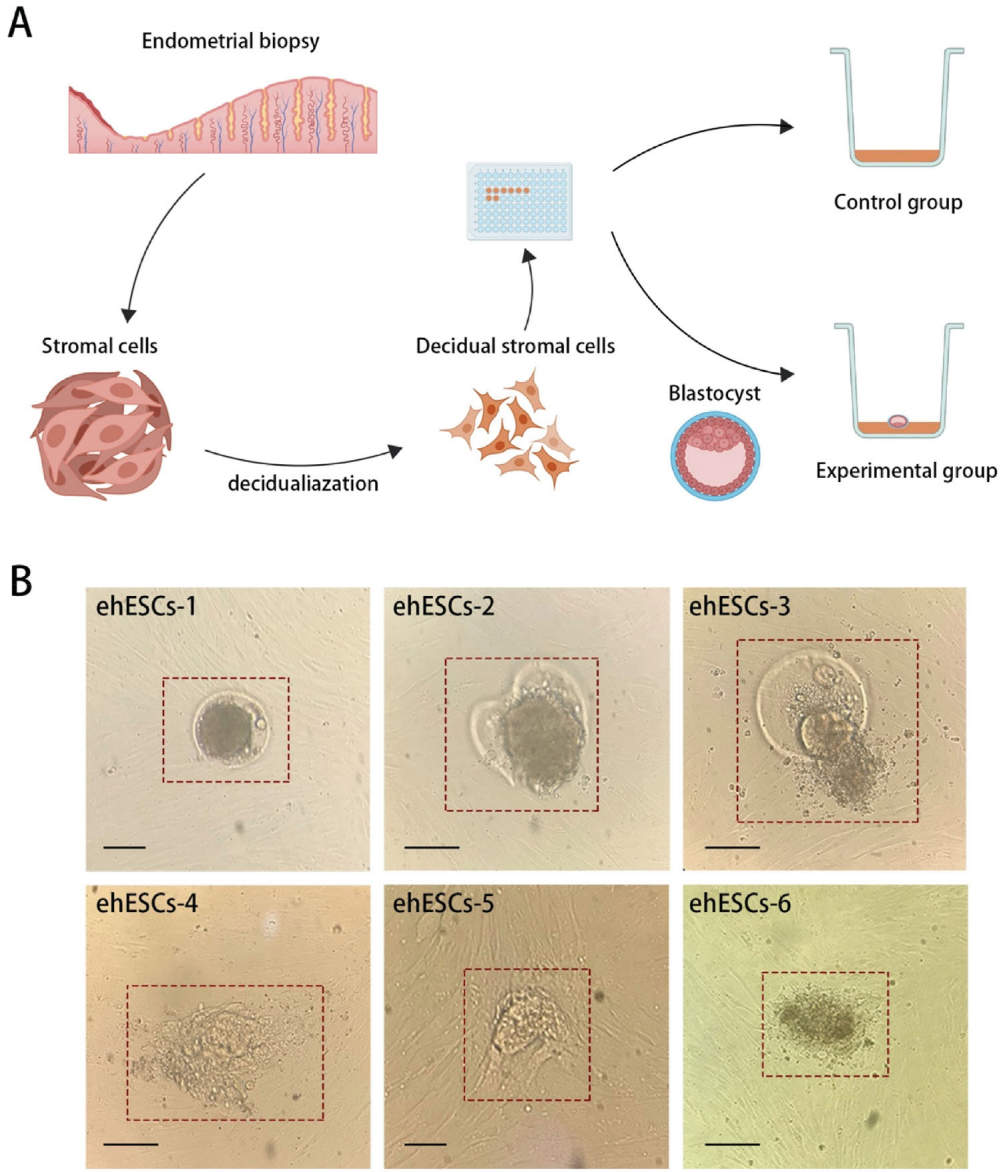
Experimental workflow of embryo invasion model *in vitro*. (A) Diagram of the experimental design. (B) Cellular morphology of embryos in the experimental group at 48 h after coculture with phESCs. The box indicates the embryos. Black horizontal scale bar: 100 μm.

The experimental design is summarized in Figure 1(A). We used phESCs within the first five generations as the bottom layer of cells and used discarded blastocysts from the same patient to eliminate experimental error attributable to differences in genetic background. Two phESCs samples (phESCS-1, phESCS-2) were used for control groups (cultured without embryos), while experimental groups included six samples of phESCs co-cultured with embryos (ehESCs-1, ehESCs-2, ehESCs-3, ehESCs-4, ehESCs-5, and ehESCs-6).

The cellular morphology of ehESCs during embryo invasion *in vitro* is shown in Figure 1(B). The red box indicates the embryos. In samples ehESCs-1, ehESCs-2, and ehESCs-3, blastocysts still retained their zona pellucida (Figure 1(B)). In ehESCs-1, embryonic cells did not climb out from the zona pellucida. In ehESCs-2 and ehESCs-3, a proportion of the embryonic cells climbed out. In samples ehESCs-4, ehESCs-5, and ehESCs-6, there was no zona pellucida, and the embryonic cells crawled out and invaded into the surrounding phESCs (Figure 1(B)).

### Global transcriptome profiling of gene expression differences between phESCs and ehESCs

**Figure 2.**
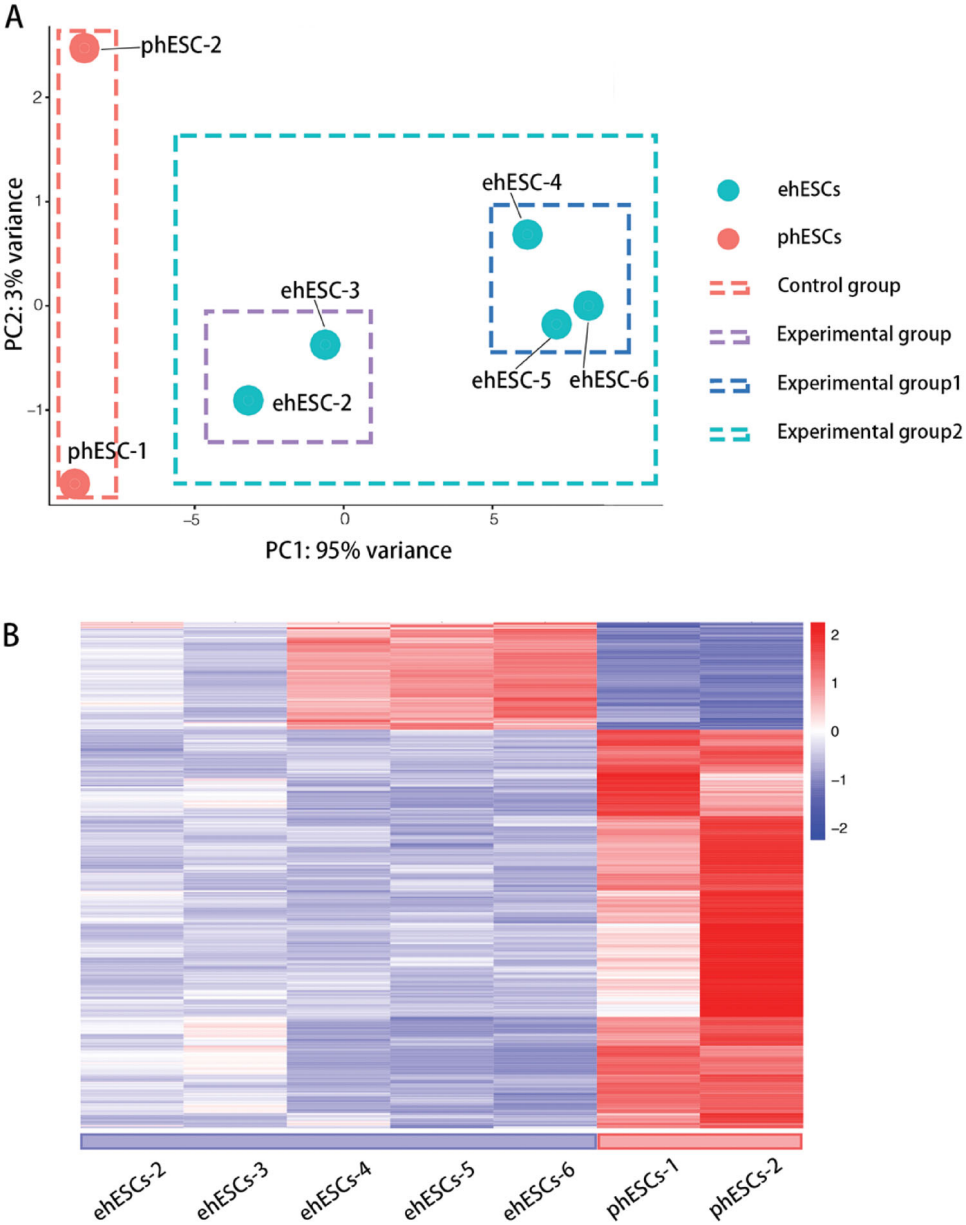
Overall transcriptomic changes between phESCs and ehESCs. (A) Principal component analysis of clusters by cell population. (B) Cluster analysis of differentially expressed genes between the phESCs and ehESCs is displayed in a heatmap. Heatmap colours represent the relative mRNA expression levels, as indicated in the colour key.

We performed RNA sequencing (RNA-seq) and analyzed the RNA-seq data from eight samples. After assessing the quality of data from all sequenced samples, the sequencing data of ehESCs-1 was found to be low-quality data. Therefore, we did not include the sequence data from ehESCs-1 in the subsequent analysis. The variability of data was checked using principal component analysis (PCA) and demonstrated that the clustering of each population was segregated from each other (Figure 2(A)).

**Figure 3.**
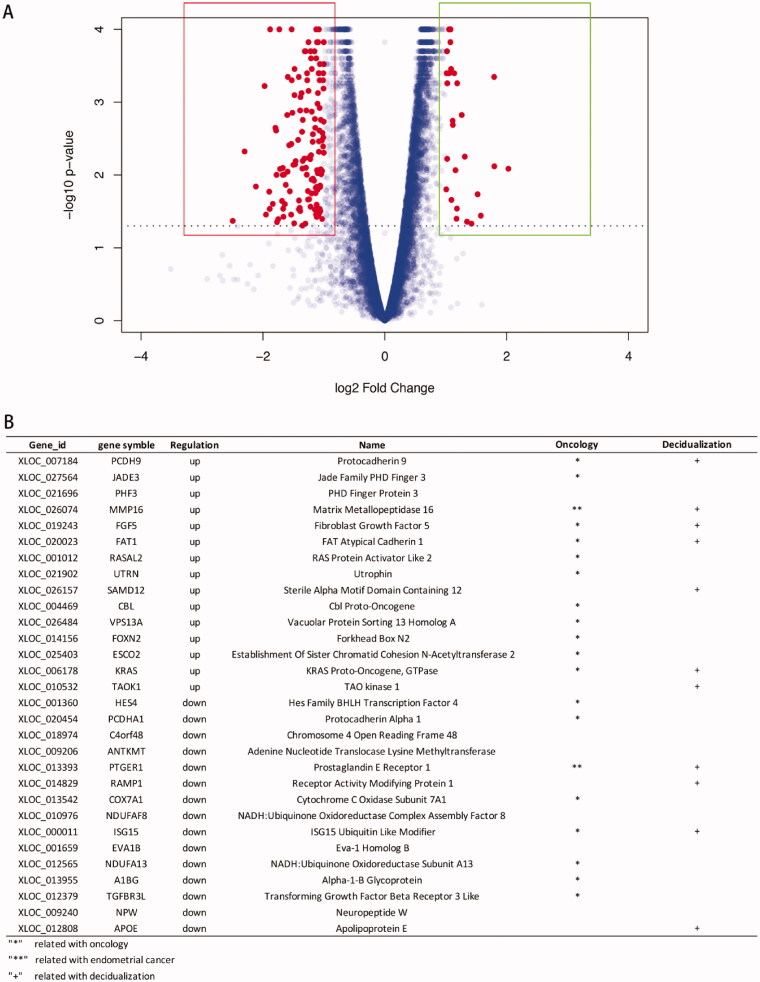
General analysis of differentially expressed genes (DEGs). (A) Volcano plot showing the distribution of up-regulated genes (dots in the right box) or down-regulated genes (dots in the left box) in phESCs and ehESCs. (B) A list of the top 15 down- and up-regulated genes.

In total, 592 genes were identified as DEGs according to similar criteria, except for |log2FoldChange| >1.5; of these DEGs, 186 were up-regulated and 406 were down-regulated between phESCs and ehESCs (Figure 2(B)). Volcano plots showing the significance of the differential gene expression *vs.* fold change are presented in Figure 3(A). Figure 3(B) displays the top 15 up-regulated and down-regulated DEGs. Two DEGs, *PTGER1* and *MMP16* have been reported in endometrial carcinoma research [31,32]. Indicated genes have been reported to be related to oncology and decidualization (Figure 3(B)).

Interestingly, 186 up-regulated genes were only differentially expressed in ehESCs-4, ehESCs-5 and ehESCs-6 (Figure 2(B)). Enrichment analysis of the 186 up-regulated genes for “cellular components” ontology terms identified association with cell junction including hemidesmosome, filopodium membrane, microtubule end, and filopodium. For “biological processes” ontology terms, the predominantly represented GO terms are cell development and differentiation-related pathway including forebrain astrocyte development, interleukin-6-mediated signalling pathway, and response to platelet-derived growth factor (Figure S1).

We further sub-grouped the samples in the experimental group (Figure 2(A)). Samples ehESCs-2 and ehESCs-3 were included in experimental group 1, while samples ehESCs-4, ehESCs-5, and ehESCs-6 were included in experimental group 2. Gene ontology analysis identified enriched GO terms in biosynthetic and cellular protein modification including ATP metabolic process, protein targeting, and organelle fission (Figure S2).

These results suggested that phESCs undergo significant transcriptomic changes after embryo attachment and invasion, and this process occurs in a stepwise fashion.

### Functional analysis of DEGs in phESCs

**Figure 4.**
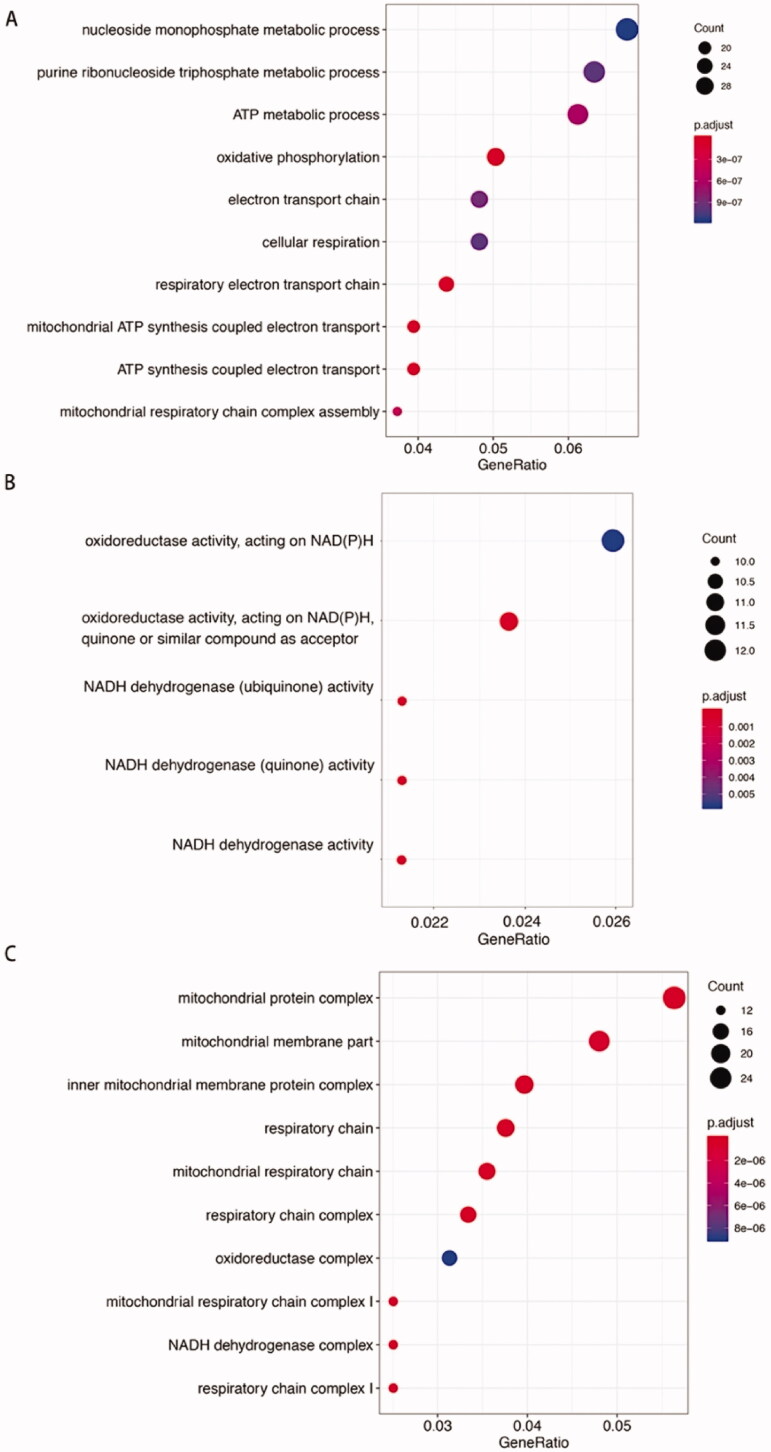
Gene Ontology enrichment analysis of phESCs and ehESCs. GO enrichment results of differentially expressed genes between phESCs and ehESCs are displayed in the bubble plots and include biological process (A), molecular function (B), and cellular component (C). The bubble colour represents the *p*-value, and the bubble size represents the number of genes in the relevant pathway (see the legend on right-hand side).

GO annotation analyses identified enrichment of DEGs in cellular respiration and mitochondrial organization including (i) mitochondrial respiratory chain complex I assembly; (ii) oxidative phosphorylation; (iii) respiratory electron transport chain. GO cellular component and GO molecular function enrichment analysis were consistent with the results of the GO biological process analysis (Figure 4).

**Figure 5.**
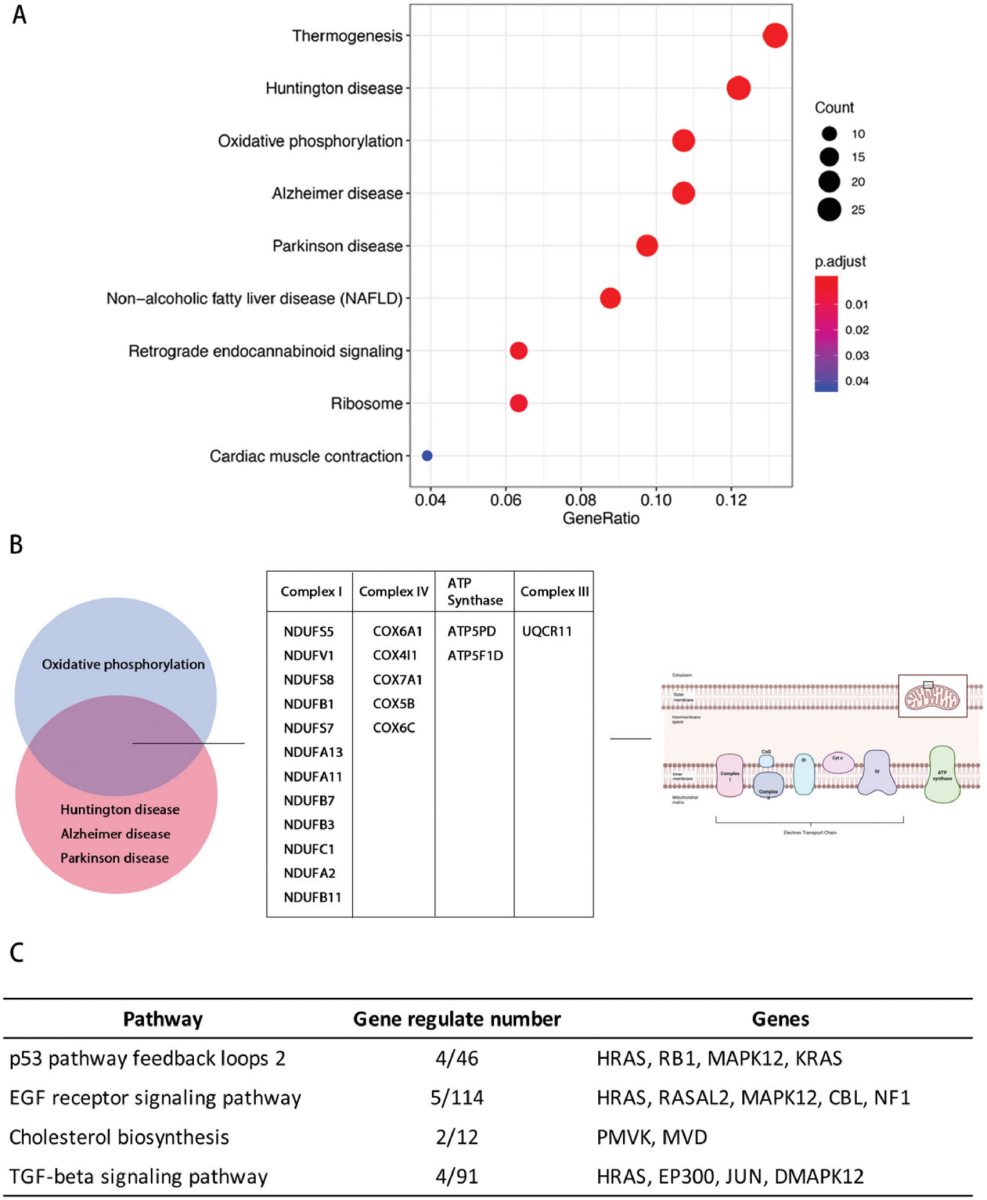
Kyoto Encyclopaedia of Genes and Genomes (KEGG) analysis of phESCs and ehESCs. (A) Bubble plot showing the top nine enriched mRNAs in KEGG pathways. The bubble colour represents the *p*-value, and the bubble size represents the number of genes in the relevant pathway (see the legend on right-hand side). (B) Twenty common genes were selected that were involved in oxidative phosphorylation, Huntington's disease, Alzheimer's disease, and Parkinson's disease KEGG pathways. The specific genes and classifications are shown in the table. The figure on the right shows the specific function of genes in oxidative phosphorylation. (C) The top four enriched PANTHER pathways and their related genes.

Analysis using the Kyoto Encyclopaedia of Genes and Genomes (KEGG) pathway database identified signal transduction pathways potentially involved in embryo invasion. The DEGs genes were mainly correlated with thermogenesis, oxidative phosphorylation, Huntington's disease, Alzheimer's disease, and Parkinson's disease (Figure 5(A)). Oxidative phosphorylation occurs in the mitochondria, and pathogenesis of Huntington's disease, Alzheimer's disease, and Parkinson's disease are closely associated with cellular respiration and energy metabolism [33–35]. Analysis of the genes common to pathways involved in Huntington's disease, Alzheimer's disease, and Parkinson's disease included important components of the electron transport respiratory chain. These data indicated that oxidative phosphorylation may be an important function involved in regulating how stromal cells participate in embryo invasion (Figure 5(B)).

Panther pathway database analysis identified differential expression of genes involved in the TP53 and EGF signalling pathways (Figure 5(C)). Activation of the p53 protein as a transcription factor could initiate programs of cell cycle arrest, cellular senescence, or apoptosis [36–38].

These results suggested that the phESCs underwent significant transcriptomic changes in oxidative phosphorylation and mitochondria organization after embryo invasion. The TP53 signalling pathway was also identified as a differentially activated pathway in phESCs.

### Protein–protein interaction (PPI) network analysis

**Figure 6.**
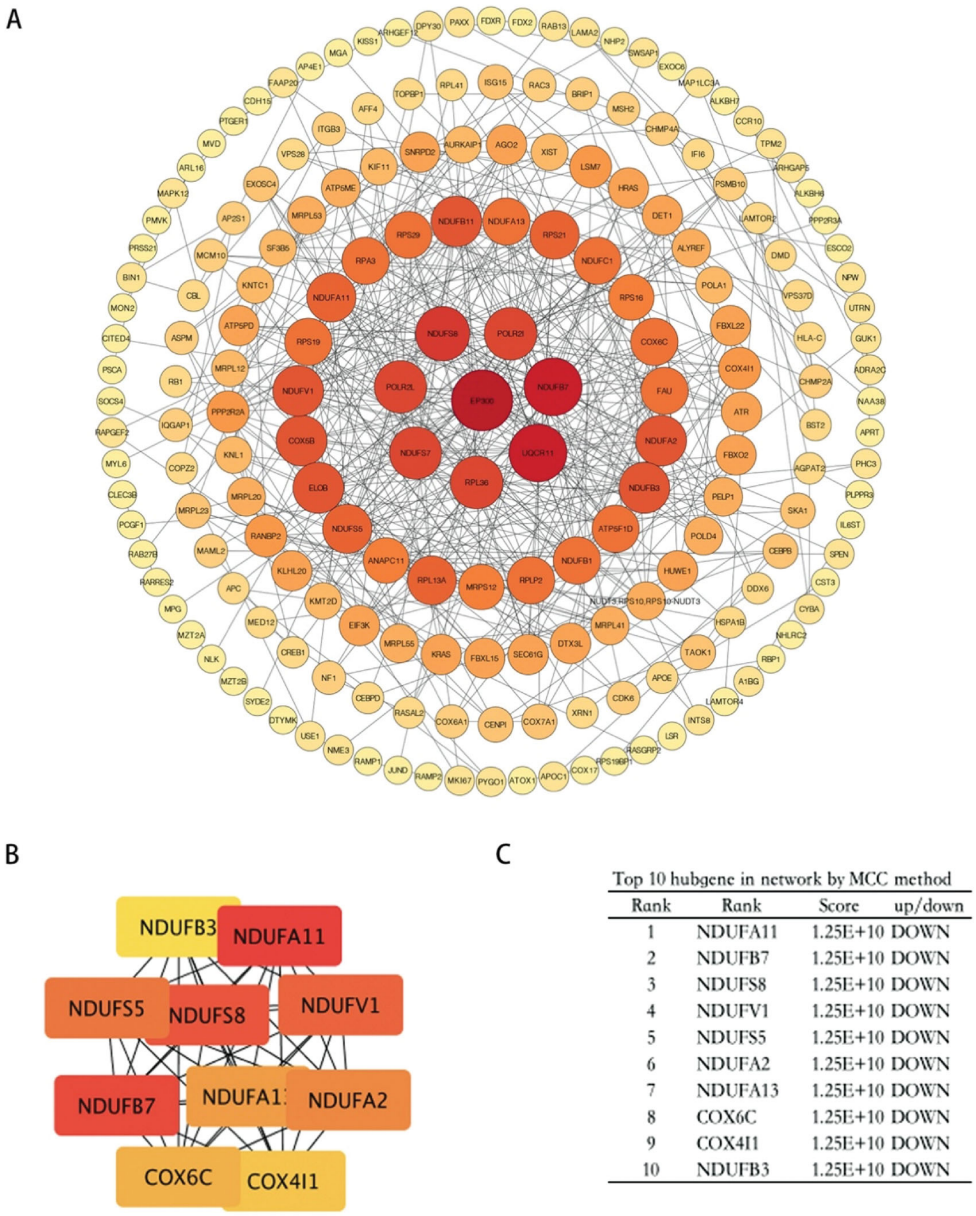
Protein-protein interaction analysis of DEGs. (A) PPI networks of the DEGs based on the STRING database analysis. The size of the node represents the degree of connectedness. The degree of a protein (node) equals the PPI interactions incident to the node in the whole network. The darker the colour and the larger the node, the higher the degree value. (B,C) PPI network of 10 hub genes from (A).

To analyze potential connectivity patterns between the transcripts in the DEG set, a protein-protein interaction (PPI) network was constructed using the STRING database. PPI analysis revealed a significant enrichment of known interactions among the DEGs (STRING PPI enrichment *p*-value, 4.69 × 10^−12^). The total DEG PPI sub-network was composed of 520 nodes and 483 edges, including 135 DEGs. Notably, functions relevant to mitochondria (cellular respiration, electron transport, and metal-binding) and oxidative phosphorylation were identified (Figure 6(A)). The top hub genes were NADH ubiquinone oxidoreductase subunit related genes, COX6C, and COX4I1, which correlated with cellular reliance on oxidative phosphorylation for energy production (Figures 6(B,C)).

In the PPI network, the gene with the highest degree of connectivity (=21) was EP300. EP300 functions as a histone acetyltransferase that regulates the transcription of genes *via* chromatin remodelling by allowing histone proteins to wrap DNA less tightly. EP300 is a significant driver gene often mutated in endometrial cancer. Down-regulation of EP300 expression shifts cellular metabolism towards oxidative phosphorylation [39]. In our results, EP300 was up-regulated, and NADH ubiquinone oxidoreductase subunit related genes were down-regulated in ehESCs, compared to phESCs.

These results suggest that human stromal cells undergo a metabolic shift characterized by attenuating oxidative phosphorylation after embryo invasion. In human stromal cells, EP300 may play a key role in regulating transcription *via* chromatin remodelling to facilitate adaptive changes for embryo invasion.

### Validation of RNA sequencing data

The differential gene expression analysis of the *in vitro* embryo invasion assay demonstrated that ehESCs had transcriptomic changes involving oxidative phosphorylation, mitochondrial organization, P53 signalling pathway, and the key node gene EP300. Next, we performed quantitative PCR to validate the differential expression of select genes between phESCs and ehESCs. We selected genes associated the most strongly with the enriched pathways and node proteins, including EP300, RB1, KRAS, COX4I1, COX6C, HRAS, MAPK12, NDUFA2, NDUFA11, and NDUFS8. EP300, RB1, and KRAS were up-regulated in ehESCs. COX4I1, COX6C, HRAS, MAPK12, NDUFA2, NDUFA11, and NDUFS8 were down-regulated in ehESCs. These changes were in agreement with the gene expression differences identified by the transcriptome sequencing (Figure S3).

## Discussion

In this study, we found that gene expression changes in phESCs during embryo invasion include genes involved in oxidative phosphorylation, mitochondria organization, and the P53 signalling pathway. We identified one candidate hub-gene, EP300, which is participating in the regulation of many genes that act during phESC decidualization and endometrial receptivity. The pathways and genes identified here may play important roles in how phESCs facilitate embryo survival and growth of.

The oxidative respiratory chain is important to the cellar function of hESCs. For example, oxidative phosphorylation is decreased in endometrial stromal cells during *in vitro* decidualization [40], and the length of the mitochondria became shortened after decidualization. In this study, we found that expression of a series of NADH ubiquinone oxidoreductase subunit related genes was significantly decreased in hESCs after embryo invasion, including mitochondrial respiratory complex I, a gene that participates in oxidative phosphorylation. Additionally, NADPH participates in the decidualization of the endometrium through the c-AMP pathway, before embryo implantation [41]. Thus, we speculate that mitochondrial oxidative respiration of hESCs decreases to adapt to embryo development after implantation.

We identified down-regulation of the TP53 signalling pathway in hESCs after embryo invasion. The TP53 signalling pathway is involved in the decidualization of stromal cells [42], and the expression of TP53 protein increased in decidualized hESCs *in vitro*. In the late secretory phase, the expression of TP53 is increased [43]. However, it is unknown why the TP53 signalling pathway is up-regulated during decidualization, then down-regulated during the embryo invasion stage.

EP300 functions as histone acetyltransferase and activates transcription by binding to transcription factors. EP300 acetylates and enhances the transcriptional activity of FOXO1, a key regulator of endometrial decidualization [44]. EP300 mediates cAMP-gene regulation by binding specifically to phosphorylated CREB protein, which participates in the decidualization of human endometrial cells [45]. These data indicate that EP300 may play an important role in upstream signalling pathways during endometrial-embryo cross-talk.

Embryo implantation is a dynamic and complex process in which embryo and endometrial cells are the key participants. The human endometrium consists of stromal cells, immune cells (mainly NK cells), and epithelial cells. To better understand the underlying mechanisms of embryo implantation, several novel methods and improved culture conditions have been developed and expanded. For instance, endometrial organoids recapitulate essential structures and functions of the receptive endometrium *in vivo*, embryo surrogates have been developed that include all three blastocyst lineages, and 3D systems have been developed, such as hydrogel-based 3D-bioprinting or micro moulding, to create microchannel systems within the tissue [27]. These more precise models can help to more accurately study the mechanisms of embryo implantation and invasion. However, the *in vitro* model employed in this study was not one of these more advanced methods and may be seen as a limitation of this study. We hope to improve upon this model in further research.

There are several other limitations in this study, including the use of discarded embryos, which may lead to different endometrial stromal cell responses, compared to high-quality embryos. For example, recurrent implantation failure is found to be related to embryo quality [46]. The discarded embryos were 4/5/6 CC grade, according to Gardner score criteria, and during the experiment, the inner cell mass and trophoblast cells of CC grade frozen embryos showed different embryo morphology after recovery (Figure 1(B)). The difference in quality between these embryos may have had an impact on the subsequent embryo invasion, which in turn affected the gene expression and gene regulatory networks of the stromal cells. Furthermore, there were only two replicates evaluated in the control group, which may have affected the quality of the sequencing data. Finally, embryo invasion is an interaction between embryo and stromal cells, and in subsequent research, we will explore the differential expression of genes and changes in the regulatory network of embryo-specific genes during embryo invasion.

Our ultimate goal is to improve embryo implantation and identify the key mechanisms underlying embryo implantation that may cause reproductive failure. In future studies, we will focus on evaluating oxidative phosphorylation, mitochondrial organization, and EP300 as novel therapeutic targets to facilitate reproductive success.

## Conclusion

Our results identify transcriptomic changes that occur in primary human endometrial stromal cells at 48 h after embryo invasion. Primary human endometrial stromal cells undergo significant transcriptomic alterations in response to embryo invasion, which occurs in a stepwise fashion. Oxidative phosphorylation, mitochondrial organization, and the P53 signalling pathway were identified as potentially having important significance in mediating the responses of primary human endometrial stromal cells during the process of embryo invasion. EP300 may play a key role in mediating adaptive changes in phESCs during embryo invasion. This study provides insights into the molecular events that occur in endometrial stromal cells during embryo invasion. The pathways identified here may help to further elucidate mechanisms underlying the embryo implantation, leading to better solutions for women experiencing reproductive failure.

## Supplementary Material

Supplemental Material

## Data Availability

The datasets used and/or analyzed during the current study are available from the corresponding author on reasonable request or online database.

## Abbreviations

hESCs: human endometrial stromal cells
phESCs: primary human endometrial stromal cells
ehESCs: human endometrial stromal cells with embryo implantation
PPI: protein–protein interaction
