# Supplementary material for: Transcriptomic analysis of human endometrial stromal cells during early embryo invasion
Source: Ann Med. 2021 Oct 13;53(1):1758–71. doi: 10.1080/07853890.2021.1988139 (PMC8519554; doi:10.1080/07853890.2021.1988139)
Supplement: Supplemental Material [file IANN_A_1988139_SM0759.zip › Embryo_informed_consent.pdf]

Beijing Chaoyang Hospital affiliated to Capital Medical University	Informed consent<br>Egg/Embryo donation on research

Name. Qian-wei Ruan　　Age. 32　　MRN. B-20190666

The patient was treated with human assisted reproductive technology in our hospital for infertility. After in vitro fertilization, only part of the eggs obtained in the treatment are suitable for transplantation or freezing. For poor quality eggs, unfertilized eggs and poor quality embryos that are not suitable for transplantation and freezing, there are the following processing methods.

1. Agree to discard
2. Agree to donate to scientific research

Patient: 2 agree

Wife (signature): Qian-wei Ruan　　Date. 21/6/2018
Husband (signature): Yu-han Liu　　Date. 21/6/2018
Physician (signature): Ying-hai Ren　　Date. 21/6/2018

首都医科大学附属
北京朝阳医院

胚胎、卵子处理知情同意书

患者 [illegible]　　年龄 32　　病案号: [illegible]

患者因患不孕症，需在我院接受人类辅助生殖技术治疗。治疗中获得的卵子经体外受精和培养后，只有部分适合移植和冷冻。对于不适合移植和冷冻的质量差的卵子、未受精卵、未形成胚胎的受精卵以及质量差的胚胎，由于没有临床治疗价值，有以下处理方式：

1. 同意丢弃。
2. 同意去标识后捐献用于医学教学与科学研究。

患者意见：2 同意

妻子（签字）[illegible]　　日期：2016 年 6 月 21 日
丈夫（签字）[illegible]　　日期：2018 年 6 月 21 日
医师（签字）[illegible]　　日期：2018 年 6 月 21 日
