# Supplementary material for: Transcriptomic analysis of human endometrial stromal cells during early embryo invasion
Source: Ann Med. 2021 Oct 13;53(1):1758–71. doi: 10.1080/07853890.2021.1988139 (PMC8519554; doi:10.1080/07853890.2021.1988139)
Supplement: Supplemental Material [file IANN_A_1988139_SM0759.zip › Endometrial_informed_consent_.pdf]

Informed consent for Clinical Research of Beijing Chaoyang Hospital Affiliated to Capital Medical University

Program: New method of endometrial receptivity measurement of human endometrial implantation window

Dear subject:

In order to further improve the clinical pregnancy rate of assisted reproduction technology and benefit the majority of infertility patients, our hospital plans to carry out research on the new method of implantation window definition and endometrial receptivity measurement. This study is an exploratory research work approved by the Ethics Committee of Beijing Chao-Yang Hospital. If you voluntarily, after reading these information, please sign and date on the last page of informed consent.

I. Research purpose

Combined endometrium function detection with traditional endometrial pathology to evaluate the accuracy of endometrial function detection, in order to future clinical application, diagnose the reason of embryo implantation failure, guide more appropriate embryo transplantation time and endometrial preparation protocol.

II. Research background

Different gene expression was reported abroad, and a few scholars in China focus on the implantation window of endometrial through mRNA markers. However, this evaluation method costs high and is time consuming with uncertain applicability. The pathologist microscopically determines the date of the uterus, and also determines the transformation of the uterus. With the help of a pathologist, about 60% of these patients adjusting transplantation strategies and luteal support programs among infertile women who failed to achieve clinical pregnancy. Endometrial morphology assessment is a low cost and efficient endometrial receptivity method.

In this study, we believe that there are individualized differences in endometrial receptivity, especially in women with repeated implantation failure who is requiring individualized treatment. Through endometrium function testing, the traditional endometrial pathology was combined with multiple recognized endometrial receptivity molecular expression tests to evaluate the accuracy of endometrial receptivity detection. We hope to conduct the future clinical application, diagnose the reason of embryo implantation failure, guide more appropriate embryo transplantation time and endometrial preparation protocol.

Ⅲ. Research process

1. How many people will participate in the study?

Sixty people will participate in this study.

2. Information and biological specimens collected in the study

We will collect your endometrium in this study. We want to be able to preserve your specimen for drug-related gene polymorphism testing several months or weeks later.Since your specimens are valuable for clinical studies, we hope to preserve your remaining specimens until the end of the study.

Your endometrial specimen will be identified by the study number number rather than your name. Only individual researchers and authorized others are able to identify your name from this code. Other researchers who conduct future research or you personally will not be able to know which studies your specimens are used for or which data are generated using your specimens.Your personal information will not be leaked unless required by the relevant law. Your specimen will exist at Beijing Chaoyang Hospital for scientific research.

This study also collects information on your external pregnancy assistance treatment regimen and pregnancy outcomes at the Reproductive Center of Beijing Chao-Yang Hospital to count baseline data, and your personal information will not be leaked.

3. Research Methods

(1) According to the exclusion criteria and pregnancy conditions, the patients were divided into experimental groups: clinical pregnancy after transplantation, and the control group: never clinical pregnancy after transplantation. There were 60 subjects each in each group. The patients performed B superovulation monitoring, with LH + for 0 days at urine or blood LH peak, HCG injection day as hCG + for 0 days, sampling time of LH/ hCG + 2 and LH/hCG + for 9 days, and blood for estrogen and progesterone levels. Based on this result, patients will adopt an individualized transplant program. Endometrial sampling for all patients was done by the same doctor, using the disposable endometrial microsampling tube absorbed from the uterine endometrium in a sterile state after excluding contraindications to endometrial operation like vaginitis and pregnancy. According to the result, the patient will adopt an individualized transplantation program, where the remaining endometrium is submitted, and the key differential expression gene and signaling pathway are found and verified by comparing the differences between endometrial tissue and single cell sequencing at different periods.

The process is guided by professional doctors, accompanied, and telephone follow-up, this study only involves endometrial tissue collection, if

you do not choose to participate in this clinical study, will not affect your reproductive center pregnancy assistance treatment, the sampling time span is not more than two months.

IV. Risk and benefit

1. **What is the risk of participating in this study?**

1) Postoperative bleeding: there may be very slight vaginal bleeding, does not affect the follow-up treatment, also will not cause any harm to the body, will heal in a short period of time;

2) pain: generally will feel no pain or very slight, will heal in the short term;

3) infection: there is almost no risk, but it can not completely exclude individual patients ' physical problems or acute attacks of original inflammation;

4) Risk of multiple endometrial samples due to insufficient intima;

5) Patients may cause discomfort during 5) sampling.

Solution: There may be a risk of infection during treatment, including: operation by experienced clinicians, strict implementation of sterility principles, strict aseptic operation, and preoperative and postoperative preventive use of oral antibiotics.

2. What does benefit from participating in the research?

Direct benefit:

1) The results of endometrial tolerance gene testing are provided free, and guide the subsequent embryo transfer program in combination with its pathological results, which is expected to achieve a successful pregnancy.

2) Good medical care is provided during treatment, priority examination, and free consultation.

Potential benefits:

1) The study had the potential to find individualized transplant programs for patients with repeated bed failures for a successful pregnancy.

2) findings can guide individualized transplantation in IVF-ET, improve pregnancy rates, and avoid unexplained repeat implant failure.

V. Use of research results and the confidentiality of personal information

With the understanding and assistance of you and other subjects, the results obtained through the project may be published in medical journals, but we will keep your research records confidential as required by law.The personal information of the study subject will be strictly confidential and will not be disclosed unless required by relevant law.If necessary, the Government

Administration and the Hospital Ethics Committee and other relevant researchers may access your information as required.

VI. Research expenses and related compensation

1. Drug and related examination costs for the Institute

You are not have to pay additional medication and examinations during the course of this study.Due to the limited funds, we cannot bear the relevant examination and treatment costs payable for your normal treatment of infertility.

2. participated in the study for compensation

For non-commercial study, you will not receive any subsidies in this study. Nor will this study interfere with the normal medical behavior you receive in the hospital.

3. Compensation / compensation after the injury

The potential risk of occurring in this study was bleeding, postoperative infection, and pain, with no serious adverse events.Bleeding and pain can be short-term self-healing. The risk rate of postoperative infection was extremely low. If postoperative infection occurs and is related to trial / study affirmation, the research group bears the cost of treating postoperative infection.

VIII. Rights of the subjects and relevant precautions

1. all of your rights

Throughout the study, you are voluntary. If you decide not to participate in this study, it will not affect the other treatments you should receive. If you decide to participate, you will be required to sign this written informed consent form. You have the right to withdraw from the study at any time at any stage without discrimination or unfair treatment, and your corresponding medical treatment and interests are not affected.

2. considerations

With scheduled ultrasound follicle monitoring and 2 endometrial microsampling according to follicle development, receive the corresponding telephone or WeChat follow-up within 3 days after the sampling, you need to provide the study doctor about your own history and current physical condition; tell the study doctor any discomfort during the study; do not take restricted drugs, food, etc.; and whether you have recently participated in other studies or are currently involved in other studies.

IX. Contact information of knowing the information

If you have any difficulties or questions about your own research data or knowledge of the findings of the study or about the study itself or its rights or

damage as a participant, you may raise any questions about this study at any time and answer answered 15210545330 With, the Ma Shuai *(For the researcher or relevant personnel, please provide a mobile phone number) to contact.*

The Ethics Committee has reviewed and approved the study, and if you have any questions related to your rights / interests, or if you want to reflect the difficulties, grievances and concerns involved in the study, or to provide comments and suggestions regarding the study, please contact Beijing Chaoyang Hospital__Ethics Committee, Tel: 010-85231484, Email: c yylunli2019@163.com.

Subject signature page

Informed consent statement:

I have been informed of the purpose, background, process, risks and benefits of this study. I have enough time and opportunity to ask questions and I am satisfied with the response to them.

I was also told who I should contact when I have problems, want to reflect difficulties, concerns, advice on the research, or want to get further information, or provide help with the research.

I have read this informed consent form and agreed to participate in this study.

I know I can choose not to attend this study or without any reason to quit this study at any time during the study.

I already know that if I am in worse condition or if I have serious adverse events or if my research doctor felt it was not in my best interest to continue in the research, he / she would decide to pull me out of research. Without my consent, the sponsor or regulator may also terminate the study during the study. If this happens, the doctor will inform me in time and the research doctor will discuss my other options with me.

Subject's signature: Xue-jiao Bao Date: 30/9/2019

(Note: If the subject is incompetent / limited, the legal agent signature and signature date.)

Contact information: NO

Signature of independent Witness: Shuai Ma Date: 30/9/2019

Independent witness contact information: 010-85231423

Investigator Signature: Shuo Han Date: 30/9/2019

Investigator Contact Information: 010-85231423

第一版，20190224 版，日期 2019 年 2 月 24 日

首都医科大学附属北京朝阳医院临床研究知情同意书

方案名称：人类子宫内膜着床窗界定及容受性衡量新方法的研究

尊敬的受试者：

为进一步提高辅助生殖临床妊娠率，造福广大不孕症受试者，我院拟开展人类子宫内膜着床窗界定及容受性衡量新方法的研究。本研究是一个探索性研究工作，经北京朝阳医院伦理委员会审查批准。如果您自愿参加，在阅读完这些资料后，请您在知情同意书的最后一页签名和签署日期。

一、研究目的

1、通过内膜功能检测，将传统内膜病理与多个公认的内膜容受性相关分子表达的检测相结合，评价内膜容受状态，评估内膜功能检测的准确度，以期未来应用于临床，诊断胚胎种植失败原因，指导更合适的胚胎移植时间以及内膜准备方案。

2、探究拮抗剂方案促排卵期间过低的血清 LH 水平对子宫内膜容受性的影响及其可能机制，更加精准的选择适合新鲜胚胎移植的患者，缩短到达妊娠时间，同时为全胚胎冷冻提供指征。

二、研究背景

既往国外报道子宫内膜容受性相关基因会发生差异性表达，国内少数学者也通过mRNA等标记物来预测子宫内膜的种植窗。但是这种评价指标花费高费时多，样本量少，适用性存在不确定性。病理学家通过对患者的子宫内膜进行显微镜下能判断子宫内膜的日期，同时判断子宫内膜的转化情况。通过病理学家的帮助，指导这部分患者调整移植策略及黄体支持方案后，反复种植失败的不孕女性中有60%左右成功临床妊娠。应用显微镜下子宫内膜形态学评估，是一种低成本高效率的子宫内膜容受性的评估方法。

通过这一研究我们认为：女性的子宫内膜容受性存在个体化差异，尤其反复种植失败的女性，需要个体化的治疗方案。通过内膜功能检测，将传统内膜病理与多个公认的内膜容受性相关分子表达的检测相结合，评价内膜容受状态，评估内膜功能检测的准确度，以期未来应用于临床，诊断胚胎种植失败原因，指导更合适的胚胎移植时间以及内膜准备方案。

三、研究过程

1. 多少人将参与这项研究？

60 人将参与本项研究。

2. 研究中收集的信息和生物标本

我们将在本研究中收集您子宫内膜。我们希望能够保存您的标本，以便在数月或数周后进行药物相关基因多态性检测。由于您的标本对于临床研究来说十分宝贵，我们希望能够保存您的剩余标本直至研究结束。

您的子宫内膜标本将以研究编号数字而非您的姓名加以标识。只有个别研究者及授权的其他人员才能够从该代码识别您的姓名。未来进行研究的其他研究者或者您个人将不能得知您标本用于哪些研究，也不会知道哪些数据是利用您的标本产生的。除非应相关法律要求，您个人信息不会被泄露。您的标本将存在北京朝阳医院用于科学研究。

本研究还会收集您在北京朝阳医院生殖中心体外助孕相关治疗方案及妊娠结局相关信息，用于统计基线数据，您个人信息不会被泄露。

2.研究方法

（1）依据纳入排除标准和患者妊娠情况，将患者分为实验组：移植后有过临床妊娠，对照组：移植后从未临床妊娠。每组各 60 名受试者。纳入的患者行 B 超下排卵监测，在自然周期中，将出现尿或者血 LH 峰值时定为 LH+0 天，将人工周期中 HCG 注射日定为 hCG+0 天，取样时间为 LH/hCG+2 和 LH/hCG+9 天，同时抽血测雌、孕激素水平。根据该结果患

者将采取个体化移植方案。所有患者的子宫内膜取样由同一名医生完成，排除阴道炎、妊娠等内膜操作禁忌症后在无菌状态下使用一次性子宫内膜微量取样管由宫底吸取子宫内膜。根据该结果患者将采取个体化移植方案，在该检查过程中，同时将取出的剩余的子宫内膜送检，通过对比不同时期内膜组织和单细胞测序的异同，找到重点的差异表达基因和信号通路并加以验证。

该过程全程有专业医生指导、陪同以及电话随访，本研究仅涉及内膜组织的采集，如果您不选择参加此临床研究，并不会影响您生殖中心的助孕治疗，取样的时间跨度不超过两个月。

四、风险与受益

1. 参加本研究的风险是什么？

1) 术中术后出血：可能会有非常轻微的阴道点滴出血，不影响后续治疗，也不会对身体造成任何伤害，短时间内会自行痊愈；

2) 疼痛：一般会没有疼痛的感觉或者非常轻微，短期会自行痊愈；

3) 术后感染：几乎不存在此风险，但也不能完全排除个别患者体质问题或原有炎症的急性发作；

4) 因内膜量不够而需要多次内膜取样的风险；

5) 取样过程中可能引起患者不适感。

解决方案：治疗过程中可能存在感染风险，应对措施包括：由经验丰富的临床医生进行操作，严格执行无菌原则，严格无菌操作，术前及术后预防性使用口服抗生素。

2. 参加研究有什么受益？

直接受益：

1) 免费提供子宫内膜容受性基因检测结果，结合其病理学结果指导后续胚胎移植方案，有望获得成功妊娠。

2) 治疗期间提供良好的医疗服务，优先检查，免费咨询。

潜在受益：

1) 这项研究对于不明原因反复着床失败的患者，有可能找到针对她们的个体化移植方案，有望获得成功妊娠。

2) 研究成果可指导 IVF-ET 中个体化移植，提高妊娠率，避免不明原因反复种植失败。

五、研究结果的使用和个人信息的保密

在您和其他受试者的理解和协助下，通过本项目研究的结果可能会在医学杂志上发表，但是我们会按照法律的要求为您的研究记录保密。研究受试者的个人信息将受到严格保密，除非应相关法律要求，您个人信息不会被泄露。必要时，政府管理部门和医院伦理委员会及其它相关研究人员可以按规定查阅您的资料。

六、关于研究费用及相关补偿

1. 研究所用的药物及相关检查费用

本研究开展过程中，您无需支付额外的药物及检查费用。由于经费有限，我们无法承担您治疗不孕症正常应缴纳的相关检查和治疗费用。

2. 参加研究的补偿

本次研究非商业行为，您参加本次研究将不会获得任何补助。本次研究也不会干预您在医院内接受的正常医疗行为。

3. 发生损伤后的补偿/赔偿

本研究发生的潜在风险为出血、术后感染和疼痛感，无严重不良事件发生。出血和疼痛

感可短期自愈．术后感染风险发生率极低．如果发生术后感染，并确定与试验/研究肯定有关，则课题组承担治疗术后感染相关费用．

八、受试者的权利和相关注意事项

1. 您的权利

在参加研究的整个过程中，您都是自愿的．如果您决定不参加本研究，也不会影响您应该得到的其他治疗．如果您决定参加，会要求您在这份书面知情同意书上签字．您有权在研究的任何阶段随时退出研究而不会遭到歧视或受到不公平的待遇，您相应医疗待遇与权益不受影响．

2. 注意事项

根据安排行超声下卵泡监测，并根据卵泡发育情况行 2 次内膜微量取样，取样后 3 天内接受相应的电话随访或微信随访，在这个过程中您需要向研究医生提供有关自身病史和当前身体状况的真实情况；告诉研究医生自己在本次研究期间所出现的任何不适；不得服用受限制的药物、食物等；告诉研究医生自己在最近是否曾参与其他研究，或目前正参与其他研究．

九、获知信息的相关联系方式

如果您对自己的研究数据，或研究结束后您希望知道本研究的发现，或您对于此项研究本身或作为参与者应享有的权利或研究相关损伤有任何困难或疑问时，您可以在任何时间提出有关本项研究的任何问题，并得到相应的解答，请通过电话 15210545330 与 马帅 *(研究者或相关人员，请提供手机号码)*联系．

伦理委员会已经审查通过该研究，如果您有与自身权利/权益相关的任何问题，或者您想反映参与本研究过程中遭遇的困难、不满和忧虑，或者想提供与本研究有关的意见和建议，请联系 北京朝阳医院 伦理委员会，联系电话：010-85231484，电子邮件：cyylunli2019@163.com．

受试者签字页

知情同意声明：

我已被告知此项研究的目的、背景、过程、风险及获益等情况。我有足够的时间和机会进行提问，问题的答复我很满意。

我也被告知，当我有问题、想反映困难、顾虑、对研究的建议，或想进一步获得信息，或为研究提供帮助时，应当与谁联系。

我已经阅读这份知情同意书，并且同意参加本研究。

我知道我可以选择不参加此项研究，或在研究期间的任何时候无需任何理由退出本研究。

我已知道如果我的状况更差了，或者我出现严重的不良事件，或者我的研究医生觉得继续参加研究不符合我的最佳利益，他/她会决定让我退出研究。无需征得我的同意，资助方或者监管机构也可能在研究期间终止研究。如果发生该情况，医生将及时通知我，研究医生也会与我讨论我的其他选择。

受试者签名：[illegible]　　　　日期：2019.9.3[illegible]

（注：如果受试者无行为能力/限制行为能力时，则需法定代理人签名和签署日期）

受试者联系方式：/

独立见证人签字：马帅　　　　日期：2019.9.30

独立见证人联系方式：010-85231423

研究者签名：[illegible]　　　　日期：2019.9.30

研究者联系方式：010-85231423
