# Supplementary material for: Transcriptomic analysis of human endometrial stromal cells during early embryo invasion
Source: Ann Med. 2021 Oct 13;53(1):1758–71. doi: 10.1080/07853890.2021.1988139 (PMC8519554; doi:10.1080/07853890.2021.1988139)
Supplement: Supplemental Material [file IANN_A_1988139_SM0759.zip › Ethical_approval_document.pdf]

Ethic Committee of Beijing Chaoyang Hospital affiliated to Capital Medical University

Ethical approval document

No. of ethic committee： 2020-5-9-4 No：2020-Science-279

<table>
<tr><td>Full Title of Study</td><td colspan="5">Transcriptome changes of human embryo endometrium before and after implantation in vitro</td></tr>
<tr><td>Project Origin</td><td>No</td><td>No.of Project</td><td>No</td><td>Target</td><td>Scientific research</td></tr>
<tr><td>Study leader</td><td colspan="5">Yuan Li</td></tr>
<tr><td>Department:</td><td colspan="5">Medical Center for Human Reproduction</td></tr>
<tr><td rowspan="9">Attached documents</td><td colspan="5">Application form for Ethical review of research projects</td></tr>
<tr><td colspan="5">Brief CV of Investigator and certificate of GCP</td></tr>
<tr><td colspan="5">Basic work of research</td></tr>
<tr><td colspan="5">Research protocol</td></tr>
<tr><td colspan="5">Informed consent</td></tr>
<tr><td colspan="5">Management system of Quality</td></tr>
<tr><td colspan="5">Risk assessment and Safety measures</td></tr>
<tr><td colspan="5">Proof of Source of funding</td></tr>
<tr><td colspan="5">Ownership of intellectual property</td></tr>
<tr><td colspan="6">In accordance with GCP Guidelines, the Organization and implementation of the Council is relatively independent</td></tr>
<tr><td colspan="6">The staff and procedures of the Ethics Committee are in accordance with Principles of GCP and national regulations</td></tr>
<tr><td>Date of approved by ethic committee</td><td colspan="5">2020/6/1</td></tr>
<tr><td>Address of the ethic committee</td><td colspan="5">F305 Beijing Chaoyang Hospital affiliated to Capital Medical University</td></tr>
<tr><td>No.of attendees： 17</td><td colspan="2">No.of avoidance (0)</td><td colspan="3">No.of abstention (0)</td></tr>
<tr><td rowspan="2">voting result</td><td colspan="5">Agree (17) Disagree (0)
Agree with necessary modifications(0)</td></tr>
<tr><td colspan="5">Reconsider votes with necessary modifications(0)</td></tr>
</table>

	Termination or suspension of test (0)
Resolutions reached during the meeting	Agree ■ Disagree □ Modification □<br>Terminate/Suspend the test □

Review opinions： None

Note:

1. .this batch will be filed with the ethics committee. If the case has different views on the organization's viability (including the qualifications and experience of the researcher, equipment and conditions, etc.), please contact the company in a timely manner.
2. Please submit the conclusion report when completing the clinical study.
3. Please submit report to the ethics committee when suspending / terminating / completing the clinical study.
4. please submit the tracking review report to the ethics committee in time according to the annual regular tracking review frequency.
5. In the event of serious adverse events and unexpected adverse events that affect the benefit ratio of the study risk, this Ethics Committee should report to you in time. in case of any modification of the clinical research programme or informed consent form and replacement of the lead researcher, the ethics committee shall be promptly notified, re-examined, and executed upon approval.
6. Any protocol deviation/violation that affects the subject's participation in the study should be reported to the ETHICS committee in a timely manner
7. The investigator should immediately report to the ERB all information that may affect the safety of the subject's live study implementation, situations that increase the risk to the subject or significantly affect the study implementation

Validity of ethical review approval	12 Months from date of issue<br>* The validity period of an ethical review approval is the period of time from the date of approval by the Ethics Committee. conducting a trial/study of this ethical review batch is effective. If the test/study is not carried out within the validity period of the ethical review approval, it is necessary to reapply for the ethical review. If a test/study is conducted within the validity period, then this ethical approval is valid.
Annual periodic follow-up review	■ Yes □ No
Frequency of annual periodic follow-up reviews	Every 12 month from the date of issue<br>* The Ethics Committee has the power to change the frequency of follow-up

Chairman(Signiture)

Date 1/6/2020

首都医科大学附属北京朝阳医院伦理委员会
科研课题会议审查批件（3.1 版）

受理号：2020-5-9-4

编号：2020-科-279

研究项目名称	人胚胎着床前后子宫内膜转录组学变化的研究				
课题来源	不适用	课题编号	不适用	评价目的	科学研究
我院项目负责人	李媛				
我院项目负责科室	生殖医学中心				
报送材料	科研项目伦理审查申请表				
	主要研究者简历及 GCP 证书复印件				
	研究工作基础				
	研究方案				
	知情同意书				
	质量管理方案				
	风险预评估及处置预案				
	经费来源证明				
	知识产权归属说明				

*依据 GCP 要求及相关法规，本伦理委员会的组织和实施相对独立
*本院伦理委员会的人员组成和工作程序是符合 GCP 原则以及国家相关规定的

审查会议日期	2020 年 6 月 1 日	
审查会议地点	北京朝阳医院门诊楼十层第一会议室	
伦理委员会出席人数（17）人	回避人数（0）人	弃权人数（0）人
投票结果	同意（17）票	不同意（0）票
	作必要修改后同意（0）票	
	作必要修改后重审（0）票	终止或暂停试验（0）票
会议决定	■同意 □作必要修改后同意 □作必要修改后重审 □终止/暂停试验 □不同意	

审查意见：

符合《中华人民共和国人类遗传资源管理条例》适用范围的研究项目，需要取得遗传办批件，并提交本中心伦理委员会备案后方可启动项目。

首都医科大学附属北京朝阳医院医学伦理委员会
