# Supplementary material for: Transcriptomic analysis of human endometrial stromal cells during early embryo invasion
Source: Ann Med. 2021 Oct 13;53(1):1758–71. doi: 10.1080/07853890.2021.1988139 (PMC8519554; doi:10.1080/07853890.2021.1988139)
Supplement: Supplemental Material [file IANN_A_1988139_SM0759.zip › Supplemental_tables_and_figures.docx]

**Figure 1**

**
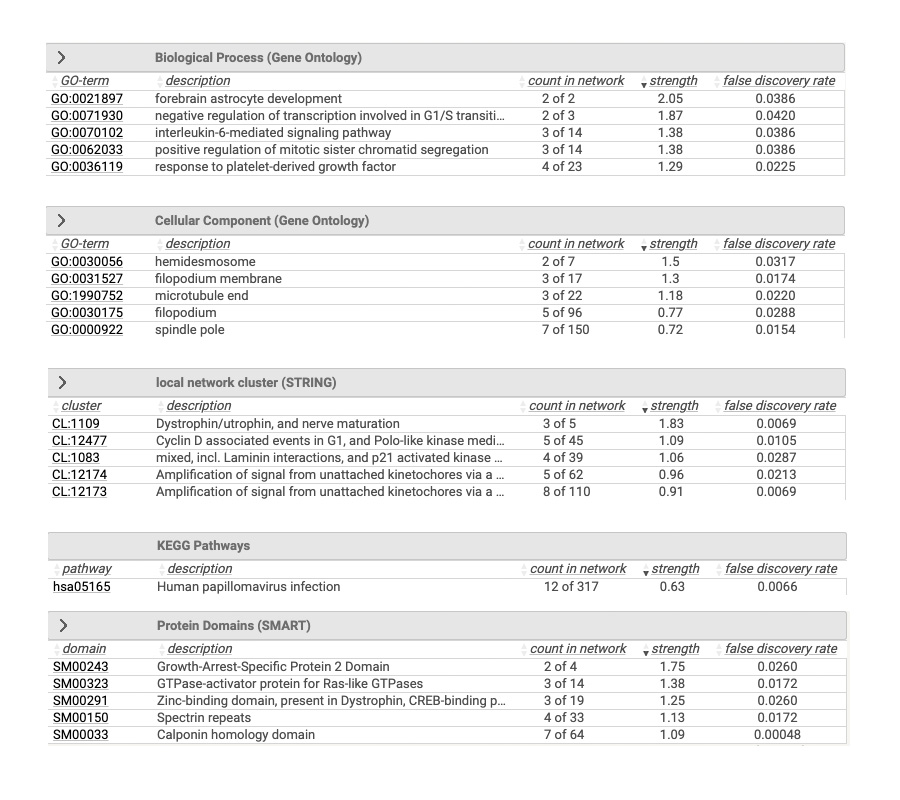
**

Figure 1: Analysis of up-regulated genes of ehESCs.

The analysis for 186 up-regulated genes was performed by STRING database, An interaction score of > 0.4 (medium confidence score) was considered significant and the functional enrichments including GO and KEGG analysis is displayed in the table.

**Table 1**

| **Gene symble** | **Sequence (5' -> 3')** | |
| --- | --- | --- |
|  | **Forward Primer** | **Reverse Primer** |
| EP300 | GCTTCAGACAAGTCTTGGCAT | ACTACCAGATCGCAGCAATTC |
| RB1 | TTGTAACGGGAGTCGGGAGA | CAGCGAGCTGTGGAGGAG |
| KRAS | TAGGCAAGAGTGCCTTGACG | CCCTCCCCAGTCCTCATGTA |
| COX4I1 | GCGGTGCCATGTTCTTCATC | GCTTGGCCACCCACTCTTT |
| COX6C | ATGCAGGGCACTTACTAGCAT | ATCCATAGCCCAAGACCAAGT |
| HRAS | GCTACGGCATCCCCTACATC | TTGTGCTGCGTCAGGAGAG |
| MAPK12 | CCCTGGATGACTTCACGGAC | GCGTGGATATACCTCAGCCC |
| NDUFA2 | CGAATCCCGACCTACCCATC | AAGAGAGAGAGGGAGGTGTCT |
| NDUFA11 | TGAACTACTTCCTCGGTGGC | CGCTATGCCAAAGTACACGC |
| NDUFS8 | GTGGACTGAGCTCTTCCGAG | TAGTTGATGGTGGCCGGTTC |


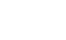


Table 1: Primers of genes.

**Figure 2**


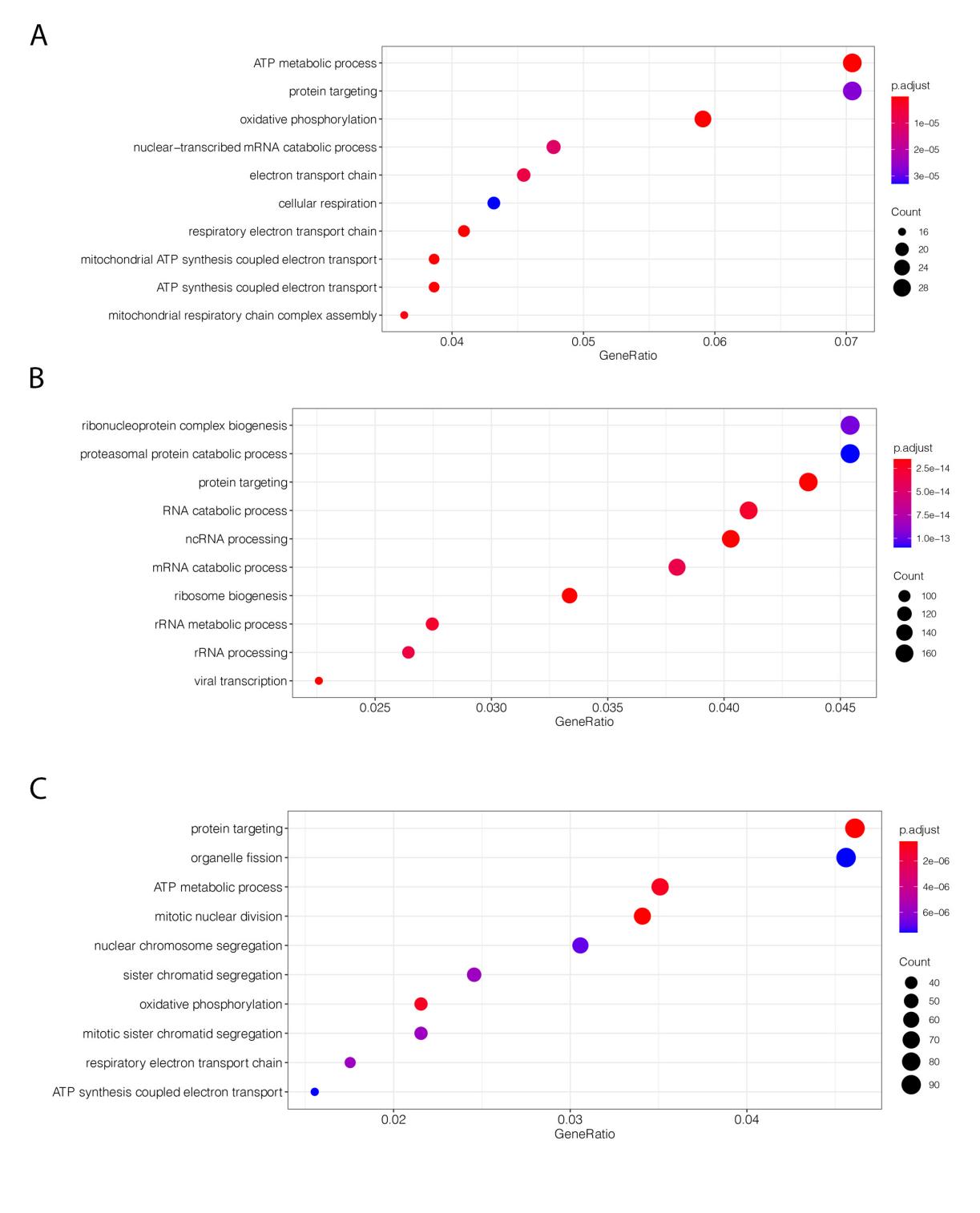


Figure 2: GO analysis between different groups.

Samples were regrouped and then compared to perform differential gene expression analysis. Then, GO analysis were performed on the obtained differential genes. GO analysis between control group and experimental group1 (A), control group and experimental group2 (B) , GO analysis between experimental group1 and experimental group2 (C).

**Figure 3**


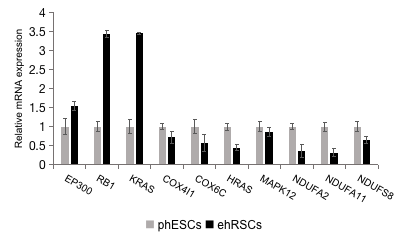


Figure 3: Quantitative real-time PCR (qRT-PCR) was employed to validate the results from high-throughput sequencing of the differentially expressed genes.

**Table 2**

| **WGC ID** | **Sanple name** | **Concentration (ng/μl)** | **volume(μl)** | **sample amounts (ng)** | **OD 260/280** | **RIN [RNA value]** | **length (bp)** | **QC result** |
| --- | --- | --- | --- | --- | --- | --- | --- | --- |
| R18042503 | HS-1 | 2.58 | 14 | 36.14 | NA | NA | 402 | A |
| R18042504 | HS-2 | 0.9 | 13 | 11.75 | NA | NA | 350 | A |
| R18042505 | HS-3 | 2.07 | 14 | 29.01 | NA | NA | 334 | A |
| R18042506 | HS-4 | 0.64 | 18 | 11.68 | NA | NA | 348 | A |
| R18042507 | HS-5 | 0.85 | 16 | 13.61 | NA | NA | 322 | A |
| R18042508 | HS-6 | 0.31 | 18 | 5.59 | NA | NA | 294 | C |
| R18042509 | HS-7 | 1.03 | 19 | 19.17 | NA | NA | 331 | B |
|  |  |  |  |  |  |  |  |  |
| QC result： | A The sample quality meets the requirements of NGS. | | | | | | | |
|  | B The samples were slightly degraded or slightly polluted; The Rin values of samples were between 7 and 8; Can be sequenced at risk | | | | | | | |
|  | C The sample quality did not meet the minimum requirements of second generation sequencing, including serious degradation, apoptosis, sample contamination, etc; The rin value of samples was less than 7; It is not recommended to use. It is recommended to change the sample | | | | | | | |
